# Application of EfficientNet‐B0 and GRU‐based deep learning on classifying the colposcopy diagnosis of precancerous cervical lesions

**DOI:** 10.1002/cam4.5581

**Published:** 2023-01-11

**Authors:** Xiaoyue Chen, Xiaowen Pu, Zhirou Chen, Lanzhen Li, Kong‐Nan Zhao, Haichun Liu, Haiyan Zhu

**Affiliations:** ^1^ Department of Gynecology Shanghai First Maternity and Infant Hospital, Tongji University School of Medicine Shanghai China; ^2^ Department of Automation Shanghai Jiao Tong University Shanghai China; ^3^ Ningbo Artificial Intelligent Institute Shanghai Jiao Tong University Ningbo China; ^4^ School of Basic Medical Science Wenzhou Medical University Wenzhou China; ^5^ Australian Institute for Bioengineering and Nanotechnology The University of Queensland St Lucia Queensland Australia

**Keywords:** artificial intelligence (AI), cervical cancer, colposcopy, precancerous cervical lesions

## Abstract

**Background:**

Colposcopy is indispensable for the diagnosis of cervical lesions. However, its diagnosis accuracy for high‐grade squamous intraepithelial lesion (HSIL) is at about 50%, and the accuracy is largely dependent on the skill and experience of colposcopists. The advancement in computational power made it possible for the application of artificial intelligence (AI) to clinical problems. Here, we explored the feasibility and accuracy of the application of AI on precancerous and cancerous cervical colposcopic image recognition and classification.

**Methods:**

The images were collected from 6002 colposcopy examinations of normal control, low‐grade squamous intraepithelial lesion (LSIL), and HSIL. For each patient, the original, Schiller test, and acetic‐acid images were all collected. We built a new neural network classification model based on the hybrid algorithm. EfficientNet‐b0 was used as the backbone network for the image feature extraction, and GRU(Gate Recurrent Unit)was applied for feature fusion of the three modes examinations (original, acetic acid, and Schiller test).

**Results:**

The connected network classifier achieved an accuracy of 90.61% in distinguishing HSIL from normal and LSIL. Furthermore, the model was applied to “Trichotomy”, which reached an accuracy of 91.18% in distinguishing the HSIL, LSIL and normal control at the same time.

**Conclusion:**

Our results revealed that as shown by the high accuracy of AI in the classification of colposcopic images, AI exhibited great potential to be an effective tool for the accurate diagnosis of cervical disease and for early therapeutic intervention in cervical precancer.

## Graphical abstract

Our connected network classifier distinguished high‐grade squamous intraepithelial lesion (HSIL) from normal and low‐grade squamous intraepithelial lesion (LSIL). And distinguishing the HSIL, LSIL and normal control at the same time.

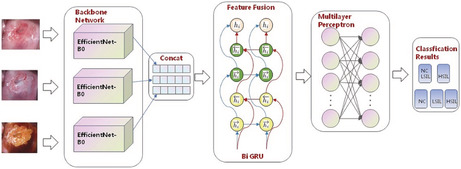

## 1 INTRODUCTION

Globally, cervical cancer is one of the most commonly diagnosed cancer among women, especially in low‐ and middle‐income countries. The World Health Organization (WHO) urged to eliminate cervical cancer globally by popularizing screening tests and the high‐risk human papillomavirus (HPV) vaccination.^1^ However, there were still an estimated 604,127 newly diagnosed cases and 341,831 related deaths in 2020.^2^ The cervical disease progresses slowly owing to a long premalignant lesion period. Thus, it provides the possibility for prevention, early detection, and treatment. The identification and intervention of precancerous lesions, including low‐grade squamous intraepithelial lesions (LSIL) and especially high‐grade intraepithelial lesions (HSIL), are regarded as the last line of cervical cancer prevention.^3^

Colposcopy was first described by Hans Hinselmann in 1925.^4^ It is acknowledged as the essential procedure for both the screening and the diagnosis of cervical lesions. Colposcopy and simultaneous cervical biopsy are the gold standards for cervical lesions diagnosis.^5^ In daily practice, women who have abnormal cytology reports or high‐risk HPV infection in screening tests should take further colposcopy examinations.^5^, ^6^ With the help of colposcopy equipment, the gynecologists observe the cervix at a magnification of 5 to 40 times. During the examination, doctors apply 3% to 5% acetic acid and Lugol's iodine solution (Schiller test) to identify potential lesions and help guide the biopsy.^7^ Colposcopy is irreplaceable in precancerous lesions diagnosis, but it still has limitations, especially the observer‐to‐observer variability due to its visual diagnostic characteristic.^4^ The concordance between the colposcopic impression and the final histopathological diagnosis is reported to be 46.9% to 65.5% .^8^, ^9^, ^10^ In addition, its accuracy is associated with the knowledge and skills of the colposcopist. Therefore, it is critically demanded to minimize the dependence on the observer experience or the subjectivity of the operator to improve diagnostic accuracy and repeatability.

In the era of big data, artificial intelligence (AI) has made impressive progress. Deep learning is a sub‐field of machine learning and is adept at data mining. It has exhibited advantages in medicine and has been reported to help provide a precise diagnosis.^11^ In early 2009, Acosta et al. employed the KNN algorithm to distinguish abnormal cervical tissues from normal ones with a sensitivity of 71% and a specificity of 59%.^12^ In the past 10 years, many studies have been published about deep learning in colposcopy diagnosis on precancerous cervical lesions, and the reported accuracy of the final validation dataset ranged from 70% to 89%.^13^, ^14^, ^15^, ^16^, ^17^

Although AI‐assisted colposcopy diagnosis exhibits advantages, it has the following shortcomings. First, the dichotomy is dominant in all the published studies with the classification confined to normal cervix (NC) vs. LSIL or worse, HSIL vs. HSIL or cancer, and LSIL vs. HSIL.^13^, ^14^, ^15^, ^16^ Secondly, there were few published studies that integrated all the original images, acetic images, and iodine images for training and validation.^18^, ^19^ Third, when it comes to the methodology, convolutional neural network (CNN) is the most commonly reported algorithm.^14^, ^18^, ^20^ Although different CNN architectures have been applied, their performance was unsatisfactory even with further manual tuning.

In this study, we used a novel deep learning‐based colposcopy assistance system to overcome the aforementioned shortcomings and greatly improved the accuracy of colposcopy precancerous lesions diagnosis. EfficientNet is a new CNN network, which is famous for its high parameter efficiency and speed.^21^, ^22^ EfficientNet‐B0 CNN model at first worked as the backbone network for colposcopy image feature extraction. It used a simple and efficient re‐combination coefficient to enlarge the CNN in a more structured way.^21^ EfficientNet‐B0 has a high‐authority network structure and an excellent feature extraction capability. Therefore, it presents potential advantages in colposcopy image classification. GRU (gate recurrent unit) is a type of recurrent neural network (RNN). GRU can greatly improve training efficiency. To our best knowledge, this is the first time that a neural network classification algorithm based on EfficientNet‐B0 and GRU is applied in colposcopy image classification. As a pioneering exploration, this study can help provide insight into the effectiveness of the application of this algorithm in this field.

All images used in clinical routine practice (original, acetic, and iodine) were collected and integrated into the model. GRU was used for further feature fusion of the original, Schiller test, and acetic acid colposcopy images. Last but not the least, in fact as part of our research that we would like to highlight, we introduced a more clinical‐convenient and doctor‐friendly triple category of colposcopy images (normal, LSIL, and HSIL).

## 2 MATERIALS AND METHODS

### 2.1 Study cohort

Information on patients and corresponding colposcopic photographs from January 01, 2017 to December 31, 2019 were retrospectively collected from Shanghai First Maternity and Infant hospital. The selection criteria were as follows: (1) age ≥ 18 years and < 70 years old, (2) not pregnant, (3) no cervical surgical history (such as previous cryotherapy, perinatal cervical cracks, laser treatment, and conization), (4) having both cytology and HPV tested results. Patient exclusion criteria were (1) poor or unclear colposcopy image(insufficient quality for reading, blurred and in which cervix was not visible), (2) presence of cervical malformations (such as double cervix), and (3) presence of cervical polyps or cervical benign neoplasms (such as uterine fibroids, inflammatory fibrous hyperplasia). To further confirm that patients in the control group without a history of other diseases, we asked about their past medical history, and review their past medical records.

All neoplastic lesions were pathologically confirmed by biopsy or conization.

All photographs were taken during colposcopy examination, before any operation or invasive procedure with a Leisegang 3Ml LED colposcopy camera (Leisegang, Germany). After applying normal saline, the transformation zone and the region of interest were evaluated after being washed with 3%–5% acetic acid and Lugol's iodine solution. A canal biopsy is regularly performed on every patient taking a colposcopy exam in our hospital. Colposcopy‐directed biopsy or conization was performed by experienced gynecologic oncologists on those patients with suspicious lesions. Colposcopy images were stored in JPEG format with a resolution of 640 × 480 pixels.

The study was performed according to the Declaration of Helsinki and was approved by the Ethical Committee of Shanghai First Maternity and Infant hospital. The EC‐approved number is KS21280. The need for informed consent was waived by the institutional review boards of the hospitals.

Totally, 612 HSIL patients, 1101 LSIL patients, and 4289 normal controls were included in our study. In total, 18,006 images were collected. We randomly divided the cohort into three sets: training set, validation set, and testing set at a ratio of 6:2:2.

### 2.2 Preprocessing

The collected images were reviewed by two gynecologic oncologists, and only the best‐quality images were selected. Since the original colposcopy image has high resolution and contains large amounts of irrelevant content, those images sent to the computer will definitely cause a large number of calculations and impair the model's performance. Two senior colposcopists extracted the suspicious lesion region of the images.

### 2.3 Deep learning‐based colposcopy image analysis

**FIGURE 1:**
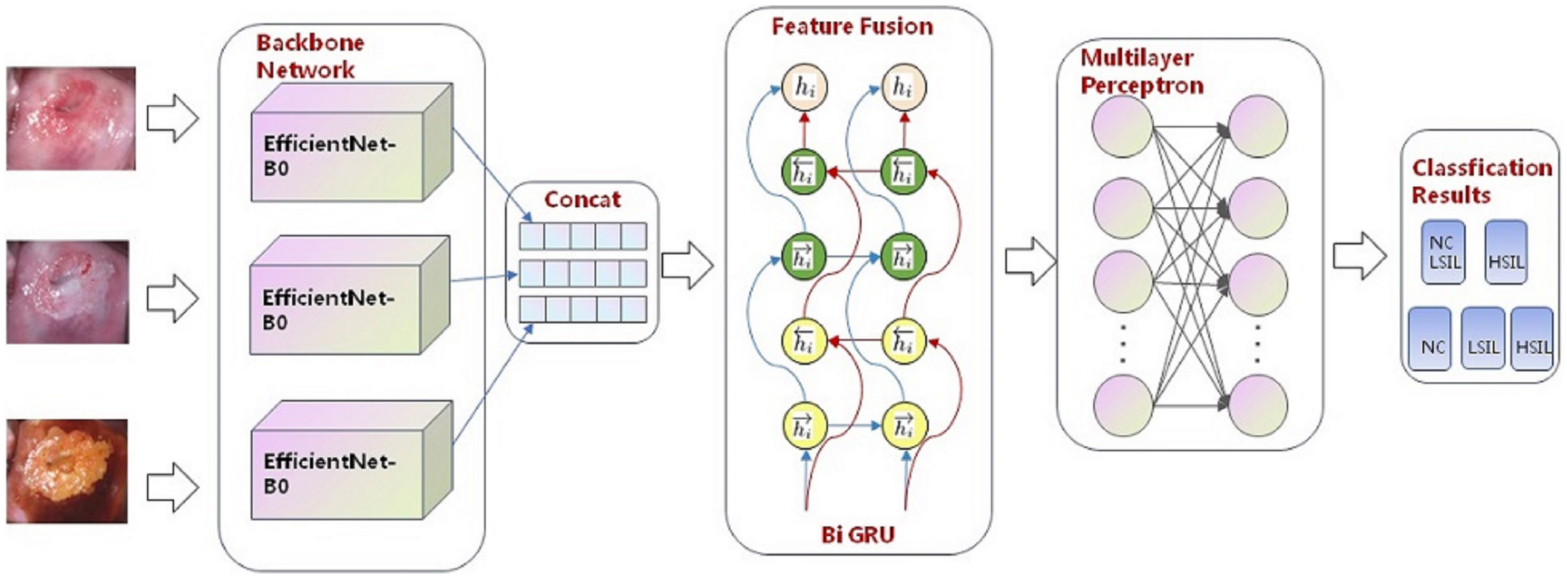
The flow chart of this study. The spatial features of the original images were extracted and resized in the model. Images were imputed, and with the data analysis, the final output is the classification results.

A neural network classification algorithm based on EfficientNet‐B0 and GRU was applied in colposcopy image classification for the first time. The detailed technical route adopted in this research is shown in Figure 1.

**FIGURE 2:**
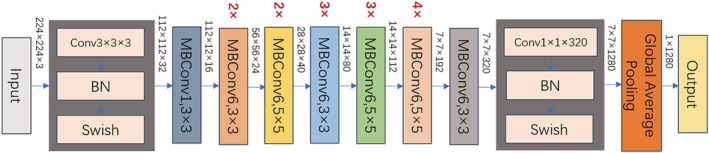
The architecture of boosted‐EfficientNet‐B0. EfficientNet first extracts the image features through its convolutional layers. The attention mechanism is then utilized to reweight the features via increasing the activation of the significant parts. Next, we performed FF on the outputs of several convolutional layers. Subsequently, the images are classified based on those fused features. Details of these methods are described in the Materials and Methods sections.

Firstly, we obtained the spatial features of the original images by EfficientNet‐B0. EfficientNet, which belongs to CNN, was used as the backbone network to extract the spatial features of the colposcopy images.^21^, ^22^ The core structure of the network was the mobile inverted bottleneck convolution (MBConv) module, which introduced the idea of squeeze and excitation network (SENet). MBConv made the model have random depth, thus reducing the time required for model training and improved the performance. EfficientNet‐B0 consists of 16 mobile flip bottleneck convolution modules, 2 convolution layers, 1 global average pool layer, and 1 classification layer. We resized the input image to 224 × 224 × 3 and performed the operations in sequence to obtain the results of the first stage as shown in Figure 2. Secondly, feature fusion was realized by contacting the features extracted from EfficientNet and 1 × 1 convolution, these layers rearrange and combine the connected features to form new features. The weight of new features is constantly updated under the constraint of the loss function to produce more satisfactory features. Finally, the concatenated long sequence features were sent to GRU to realize integrated feature extraction. After that, the integrated features were sent to the classification layer to realize the classification algorithm.

The specific characteristics of each part of EfficientNet‐B0 with GRU were shown in Figure 2. The detailed procedures are described in detail as follows.

1. The colposcopy image was first sent to the model for resizing to 224 × 224 × 3, and then to the EfficientNet layer for spatial feature extraction. The input size of EfficientNet was 224 × 224 × 3 and the output features with three dimensions at 7 × 7× 320. After batch normalization (BN), swish activation function, and global average pooling, the final output dimension of EfficientNet‐B0 is 1 × 1280.
2. Three feature maps extracted from three types of original images were sent to the features fusion function layers to achieve the features’ fusion. The torch.cat() function was used to splice these features maps and realize the final output of the spliced feature map size of 3 × 1280.
3. The concatenated long sequence features were sent to bi‐GRU to realize the integrated feature extraction, with an output of bi‐GRU module at 2 × 256 dimensions.
4. The feature map was entered into the first fully connected layer, which consisted of several fully connected layers containing the hidden layer units of 512 × 256 × 128 × 64 × 32 × 16 × 8. After that, it was sent to Softmax classification layer to realize the classification algorithm of the colposcopy images.

### 2.4 Main outcome measures and statistical analysis

To evaluate the model performances, the accuracy, sensitivity, and specificity measures were performed. The receiver operating characteristic curve (ROC) was also calculated.^23^ In the current ROC analysis, the outcomes were binary outcomes. However, with the development of machine learning and data mining, the prediction of multi‐classification is becoming more and more common, and ROC analysis is a common method to evaluate the prediction effect of models. Therefore, ROC analysis of multi‐classification outcomes is really necessary. The micro average method provided by Python's sklearn package can be used for ROC analysis of multi‐classification outcomes, and the results of multi‐classification can be transformed into the manifestation of two classifications.^24^, ^25^ Statistical analysis was performed using Python 3.7. This study was implemented on the PyTorch framework, and the results were run on NVIDIA RTX 3080ti GPU. Data are updated online at https://github.com/haichunliu/EfficientNet‐B0.

## 3 RESULTS

### 3.1 Patient demographics

Characteristics of the patients are summarized in Table 1. Totally, 6002 women were included in the study, among them 612 women being histologically confirmed HISL, 1101 being LSIL patients, and 4289 as normal controls. The cohort study population enrolled in our study was a random sample of adult women in Shanghai First Maternity and Infant Hospital, ranging in age from 18 to 70 years. There is no significant difference in the mean age among the three groups. Most of the women had more than 2 gravidities and had no more than two parties. Condom contraception was not performed in most cases. Most cases were infected with carcinogenic HPV types excluding HPV16 and 18.

**TABLE 1:** Colposcopy and biopsied characteristics of the patients

Patient characteristics	Normal (*n* = 4289)	LSIL (*n* = 1101)	HSIL (*n* = 612)
Age (years)			
Mean	40.3	37.9	41.2
Range	18–70	18–70	19–80
Gravidity			
≤2	1972	463	284
>2	2317	638	328
Parity			
≤2	4066	1048	543
>2	223	53	69
Condom contraception			
Yes	1475	362	205
No	2814	739	407
Enrollment Pap result			
Normal	1881	263	49
ASCUS	1814	349	109
LSIL	507	385	176
HSIL(HSIL,ASC‐H)	87	104	278
Enrollment HPV result			
Negative for carcinogenic types	1430	102	30
HPV16, 18	772	208	279
Positive (not HPV16,18)	2087	791	303

Abbreviations: ASC‐H, Atypical squamous cell cannot exclude HSIL; ASCUS, Atypical squamous cell of undetermined significance; HPV, Human papillomavirus; HSIL, High‐grade squamous intraepithelial lesions; LSIL, Low‐grade squamous intraepithelial lesions; NC, Normal cervix.

**FIGURE 3:**
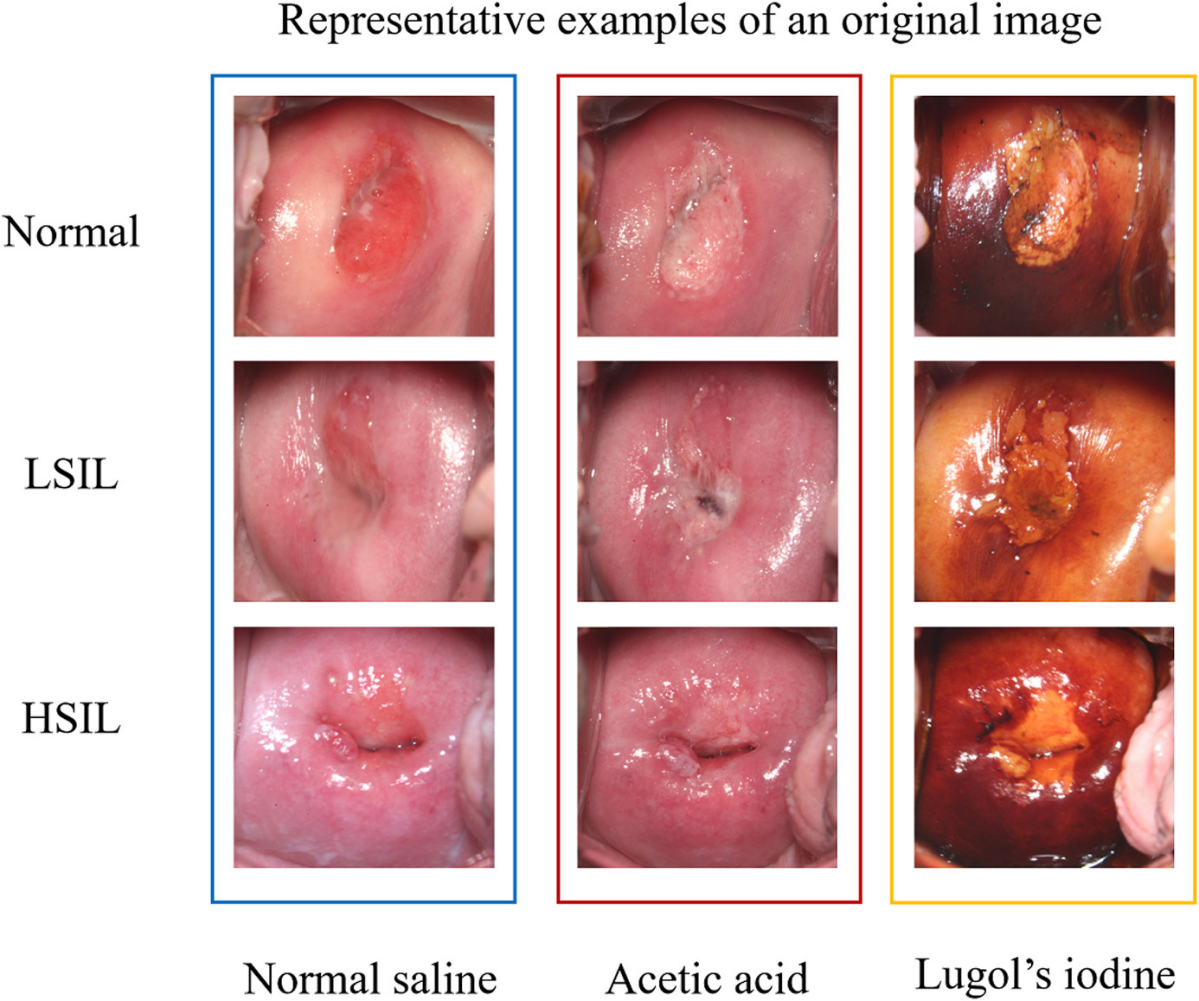
Representative examples of the original images. Every patient had three pictures: normal saline, acetic acid, and Lugol's iodine solution.

All the patients had three pictures: normal saline or original, acetic acid, and Lugol's iodine solution. The representative examples of original images are shown in Figure 3.

### 3.2 Prediction patients between HSIL− and HSIL+

**FIGURE 4:**
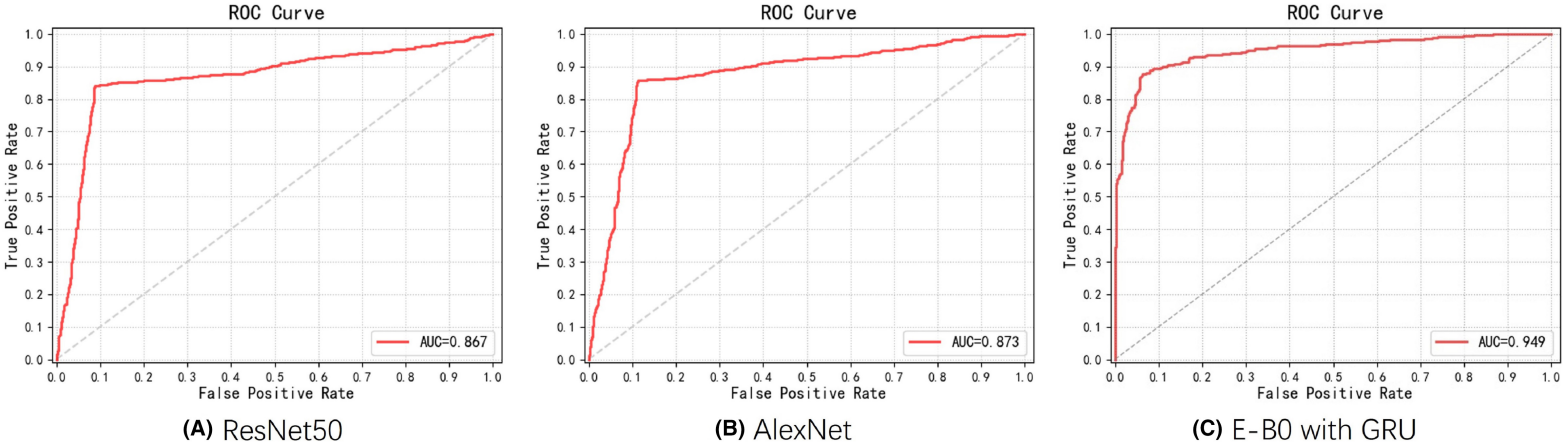
ROC curve of the dichotomous classification model (LSIL+ NC vs. HSIL)by Resnet50, AlexNet, and E‐B0 with GRU.

We randomly divided the cohort into three sets: training set, validation set, and testing set at a ratio of 6:2:2. The results came from the test dataset which is part of our cohort. In the dichotomous classification model, we screened the HSIL from the normal control and the LSIL. Those three different states of the cervical images were entered into the neural network model for the performance tests. We used classical neural networks such as AlexNet and Res Net50 as positive control. The new model based on EfficientNet‐B0 with GRU has an accuracy of 90.61% and a sensitivity of 93.6% and a specificity of 87.6%. As shown in Table 2, the new model EfficientNet‐B0 with GRU showed significantly better performance than AlexNet and Res Net50. The ROC curve was then established (Figure 4), revealing that EfficientNet‐B0 with GRU has high sensitivity and accuracy in classifying HSIL− and HSIL.

**TABLE 2:** Accuracy, sensitivity, and specificity of LSIL+ NC vs. HSIL classified by our dichotomous classification model incomparition with two other neural network architectures

Model	Accuracy (%)	Sensitivity (%)	Specificity(%)
AlexNet	83.95	87.42	91.12
Res Net50	87.35	85.62	88.79
E‐B0 with GRU	90.61	93.6	87.6

Abbreviations: HSIL, High‐grade squamous intraepithelial lesions; LSIL, Low‐grade squamous intraepithelial lesions; NC, Normal cervix.

### 3.3 Prediction NC, LSIL, and HSIL at the same time

**FIGURE 5:**
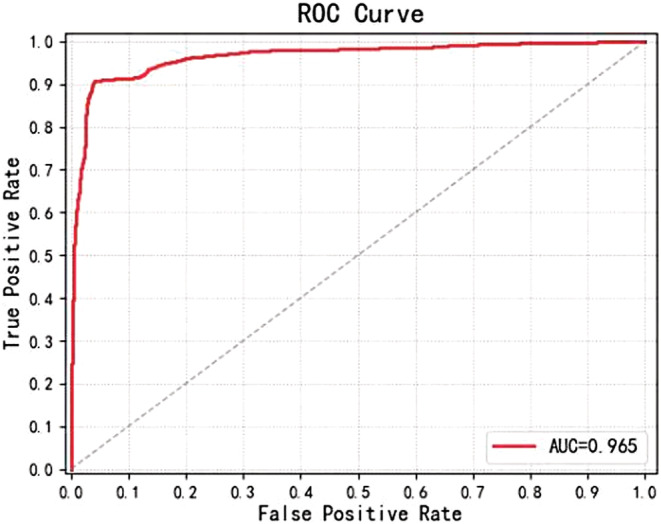
ROC curve of the three‐classification model (NC Vs. LSIL Vs. HSIL by E‐B0 with GRU.

The same model was further applied to a triple category of colposcopy images. We found that it automatically identified the normal, LSIL, and HSIL simultaneously. Similar to the aforementioned dichotomous classification model, we compared our model with popular neural network architectures to validate the results. As shown in Table 3, the EfficientNet‐B0 model with GRU had an accuracy of 91.18%, which was significantly higher than those of other neural network architectures. Table 3 lists the sensitivity and specificity of the EfficientNet‐B0 model with GRU for the normal control, LSIL, and HSIL analyzed. The sensitivity of the normal control, LSIL, and HSIL is 90.82%, 90.00%, and 81.20%, and the specificity is 97.11%, 90.73%, and 93.33%, respectively. In the ablation experiment (Table 4), we compared the accuracy of EfficientNet‐B0 models with and without the GRU component. We observed that EfficientNet‐B0 with GRU performed better than EfficientNet‐B0 only (an accuracy of 91.18% vs the accuracy of 88.32%). The ROC of the three classifications shown in Figure 5 suggested that the new model EfficientNet‐B0 with GRU has high sensitivity and specificity in classifying normal control, LSIL, and HSIL.

**TABLE 3:** The accuracy of NC Vs. LSIL Vs. HSIL is classified by our model compared with that of two other neural network architectures, and the sensitivity and specificity for normal control, LSIL, and HSIL individually of the EfficientNet‐B0 with GRU model

Model	Accuracy (%)	
AlexNet	85.12	
ResNet50	86.16	
EfficientNet‐B0 with GRU	91.18	

EfficientNet‐B0 with GRU	Sensitivity (%)	Specificity (%)
Normal control	90.82	97.11
LSIL	90.0	90.73
HSIL	81.2	93.33

Abbreviations: NC, Normal cervix; LSIL, Low‐grade squamous intraepithelial lesions; HSIL, High‐grade squamous intraepithelial lesions.

**TABLE 4:** The predicated accuracy of the EfficientNet‐B0 with and without GRU

Model	Accuracy (%)
Only‐EfficientNet‐B0	88.32
EfficientNet‐B0 with GRU	91.18

## 4 DISCUSSION

AI algorithms have been used for medical image analysis and have achieved great progress in the past few years. Meanwhile, traditional binocular colposcopy is gradually replaced by digital colposcopy, owing to the technological development in digital science. The AI‐guided digital colposcopy has been developed.^4^ In the present study, we developed a deep learning‐based colposcopy system composed of EfficientNet‐B0 and GRU models. Our model can diagnose precancerous lesions of the cervix and obtained an accuracy of 90.61% in differentiating normal and LSIL Vs. HSIL and 91.18% accuracy in distinguishing healthy control, LSIL, and HSIL.

CNN, which is widely used to extract image features or directly complete classification and detection tasks, has been widely used in clinical medicine.^26^ However, data sets in the medical field have the characteristics of few labeled samples and unnatural images, and it is difficult for conventional CNN to cope with such challenges. EfficientNets uses a simple and efficient composite coefficient to magnify CNN in a more structured way.^27^ Unlike traditional methods that arbitrarily scale network dimensions, such as width, depth, and resolution, this method uses a series of fixed scaling coefficients to uniformly scale network dimensions, which can maximize the overall network performance. Although the GRU network increases the complexity of the network, it introduces the relationship between image sequences in the time dimension, which can extract the changing trend of image sequences, increase the receptive field of EfficientNets network, and improve the generalization performance of the model.^28^

Cervical lesions were classified as LSIL or HSIL according to the lower anogenital squamous terminology.^3^ Generally, HSIL is more likely to develop into cervical cancer and should take more attention and follow‐up with regular visits.^29^ Thus, it is important to screen out HSIL patients to prevent them from developing cervical cancer. Most of the LSILs are regarded as a transient expression of HPV infection and regresses spontaneously.^30^ However, there is still a part of LSIL that progresses to HSIL.^30^ The diagnostic accuracy of traditional colposcopy in detecting HSIL or worse (HSIL+) ranged only from 30 to 70%.^31^ The sensitivity of colposcopy diagnosis for high‐grade lesions/carcinoma (HSIL+) was 71.6% in a study from Tianjin, China^8^ and 80.9% in a recent study from Chengdu, China.^9^ A published study from Japan also showed accuracy of 79.7%, accompanied by a sensitivity of 83.1% and a specificity of 77.3% for predicting pathological HSIL/LSIL by gynecologic oncologists.^13^ The timely intervention for those HSIL patients is especially important, thus we simplified our classification system to distinguish HSIL from normal tissue and LSIL. We used the efficient Net‐B0 and GRU‐based deep learning methods and achieved a high accuracy at 90.61%, with 93.6% sensitivity and 87.60% specificity. Apparently, our accuracy is higher than that of the traditional colposcopy. Our studies have revealed that the deep learning‐based colposcopy tool has exhibited superiority when compared with traditional colposcopy diagnosis.

Several scientists have reported their study results in colposcopy classification using the CNN‐based deep learning method. As early as 2014, Simoes et al. classified 170 colposcopy images using artificial neural networks (ANN) and obtained an accuracy of 72.15%.^17^ Japanese scholar Miyagi et al. developed a CNN‐based AI method on colposcopy image analysis and achieved an accuracy of 82.3%, with a sensitivity of 80%, and specificity of 88.2% in the classification of LSIL and HSIL+.^13^ The Korean scientists Cho and colleagues applied deep learning methods to distinguish HSIL and LSIL based on the pretrained CNN. They achieved an accuracy of 74.7 ± 1.8% and got a mean AUC value of 0.708 ± 0.024.^14^ Recently, with a computer‐aided diagnosis system based on residual neural network (ResNet) model, Liu et al reported an accuracy of 88.6% in classifying NC and LSIL+, and an accuracy of 80.7% in classifying HSIL− and HSIL+.^16^ Several other studies on AI‐based colposcopy classification have been reported. Hu et al. used the Faster region‐based CNN and obtained an AUC value of 0.91 in detecting precancer/cancer.^15^ Song et al. reported an accuracy of 89%, and a sensitivity of 83.21% for CIN2+ detection based on images and 3 clinical information (Cytology, HPV infection, and age).^32^ Li et al. applied a graph convolutional network with edge features (E‐GCN) on LSIL+ identification and achieved a classification accuracy of 78.33%.^33^ Although all the published studies have shown great potentiality in AI‐based colposcopy classification of precancerous cervical lesions, none of them achieved a diagnostic accuracy of over 90%. Here, our study has shown its superiority, with several points highlighted below: (1) Two senior colposcopists confirmed the suspicious cervical lesion together. (2) Three types of images: regular saline colposcopic images, post‐acetic‐acid images, and post‐iodine images were included for the classification, which could reveal the cervical lesions in multi‐dimensions. (3) We are the first to use a new and very potential CNN, the EfficientNet, in colposcopy classification. The EfficientNet model has random depth, which can reduce the time required for model training to improve its performance.

In our practical clinical cases, LISL cannot be ignored because approximately 20% of LSIL are persistent, and 10% will progress to HSIL in the following years.^34^ However, LSIL managements from both the patients and doctors are insufficient. In clinic, it is also important to distinguish LSIL from normal or transient HPV infection. In this study, to make our algorithm closer to the real clinical situation, we further proposed a three‐classification model to distinguish healthy control, LSIL, and HSIL. Surprisingly, we have achieved an accuracy of 91.9%. Furthermore, the new scaling neural network model had an excellent performance, and we are the first to introduce a clinically convenient and doctor‐friendly triple category for colposcopy images (normal, LSIL, and HSIL).

AI has been studied in cervical disease by many scientists.^18^, ^35^, ^36^, ^37^ The AI system can be used as a decision support tool in the diagnosis of cervical cancer, especially in low resources settings, where the expertise and the means are limited.^38^ The AI system can also be used as a prognosis predictor. The AI prognostic prediction support system was developed by a Japanese scientist. Because the HSIL occupancy in the uterovaginal area was significantly correlated with CIN2 patients’ prognosis. The number of high‐grade lesions in 12 segments detected by an AI‐based system was comparable to that detected by senior colposcopists. The overall correct response rate of the AI algorithm for detecting high‐grade lesions was 89.7%.^39^

The AI system we developed here has shown satisfactory accuracy in distinguishing the normal control, LSIL, and HSIL based on the retrospective analyses. But several limitations are still present, which need to be improved in the future study. Thus, we will perform further prospective studies to validate the model. Second, all the samples were collected from our institution, which might have some biases. In addition, in this study, we only focused on analyzing the cervical neoplasia lesions. Other miscellaneous situations including polyps, stenosis, and condyloma need to be explored in the future. Finally, the basic clinical features included in the study were not sufficient, especially for a high‐quality study. Thus, we plan to include more parameters such as smoking history, age of first sexual debut, and number of sexual partners in the following study.

## 5 CONCLUSIONS

Here, we developed and optimized a deep learning‐based system aiming to classify cervical squamous epithelial lesions by analyzing the colposcopy images. In this system, we achieved 90.61% accuracy at distinguishing the HSIL− and normal tissue and LSIL, and 91.18% accuracy at distinguishing healthy control, LSIL and HSIL. This AI‐aided system produced a diagnostic accuracy that is greatly superior to the traditional colposcopy analysis. We believe that AI‐guided digital colposcopy may create a novel cervical cancer screening model, substantially improve the accuracy and repeatability of colposcopy, upgrade the efficiency in the identification of precancerous lesions, and help to accelerate the elimination of global cervical cancer.

## AUTHOR CONTRIBUTIONS

**xiaoyue chen:** Writing – original draft (lead); writing – review and editing (equal). **Xiaowen Pu:** Formal analysis (equal); project administration (equal). **Zhirou Chen:** Formal analysis (equal); project administration (equal). **Lanzhen Li:** Data curation (equal); methodology (equal); software (equal). **Kong‐Nan Zhao:** Conceptualization (supporting); writing – review and editing (equal). **Haichun Liu:** Software (equal); supervision (equal); validation (equal); visualization (equal). **Haiyan Zhu:** Conceptualization (lead); supervision (equal); writing – original draft (supporting).

## Data Availability

The data presented in this study are available on request from the corresponding author. The data are not publicly available because are propriety of Shanghai First Maternity and Infant Hospital, Tongji University School of Medicine.
